# Multi-omic molecular comparison of primary versus metastatic pancreatic tumours

**DOI:** 10.1038/s41416-019-0507-5

**Published:** 2019-07-11

**Authors:** Gagandeep Brar, Edik M. Blais, R. Joseph Bender, Jonathan R. Brody, Davendra Sohal, Subha Madhavan, Vincent J. Picozzi, Andrew E. Hendifar, Vincent M. Chung, David Halverson, Sameh Mikhail, Lynn M. Matrisian, Lola Rahib, Emanuel Petricoin, Michael J. Pishvaian

**Affiliations:** 1 Lombardi Comprehensive Cancer Center, Georgetown University, Washington, DC USA; 2 Perthera Inc, Mclean, VA USA; 3 Sidney Kimmel Cancer Center, Thomas Jefferson University, Philadelphia, PA USA; 4 Cleveland Clinic, Cleveland, OH USA; 5 Innovation Center for Biomedical Informatics, Georgetown University Medical Center, Washington, DC USA; 6 Virginia Mason Hospital and Medical Center, Seattle, WA USA; 7 Cedars-Sinai Medical Center, Los Angeles, CA USA; 8 City of Hope, Duarte, CA USA; 9 Mark H. Zangmeister Cancer Center, Columbus, OH USA; 10 Pancreatic Cancer Action Network, Manhattan Beach, CA USA

**Keywords:** Predictive markers, Pancreatic cancer

## Abstract

**Background:**

Molecular profiling is increasingly used to match patients with metastatic cancer to targeted therapies, but obtaining a high-quality biopsy specimen from metastatic sites can be difficult.

**Methods:**

Patient samples were received by Perthera to coordinate genomic, proteomic and/or phosphoproteomic testing, using a specimen from either the primary tumour or a metastatic site. The relative frequencies were compared across specimen sites to assess the potential limitations of using a primary tumour sample for clinical decision support.

**Results:**

No significant differences were identified at the gene or pathway level when comparing genomic alterations between primary and metastatic lesions. Site-specific trends towards enrichment of MYC amplification in liver lesions, STK11 mutations in lung lesions and ATM and ARID2 mutations in abdominal lesions were seen, but were not statistically significant after false-discovery rate correction. Comparative analyses of proteomic results revealed significantly elevated expression of ERCC1 and TOP1 in metastatic lesions.

**Conclusions:**

Tumour tissue limitations remain a barrier to precision oncology efforts, and these real-world data suggest that performing molecular testing on a primary tumour specimen could be considered in patients with pancreatic adenocarcinoma who do not have adequate tissue readily available from a metastatic site.

## Background

Pancreatic cancer is the third leading cause of cancer-related death, with a median overall survival of <12 months. In 2018, there were an estimated 554,400 new cases and 44,330 deaths.^1^ By 2030, pancreas cancer is projected to become the second leading cause of cancer-associated mortality.^2,3^ Approximately 15–20% of patients have resectable disease at presentation, providing the only chance of curative treatment; however, only 28% survive up to 5 years.^4^ A majority of these patients ultimately develop distant recurrence and outcomes for unresectable or advanced-stage disease remain poor.^5^ For patients with metastatic pancreatic cancer, combination systemic therapies with gemcitabine/nab-paclitaxel or FOLFIRINOX have resulted only in modest improvements in outcome.^6,7^

In pancreatic cancer, whole-genome sequencing reveals a complex mutational landscape.^8^ Activating mutations in *KRAS* occur almost universally, followed by inactivation of *TP53*, *SMAD4* and *CDKN2A*. The prevalence of additional mutations involved in carcinogenesis are less common, reflecting interpatient heterogeneity.^8^ Currently, the molecular biology that links genetic changes to the aggressive nature of pancreatic cancer remains poorly defined.^5^ These mutations allow for a survival and growth advantage to pancreatic cancer, as demonstrated by invasive and metastatic phenotypes that are resistant to conventional treatment strategies.^5^

In certain tumour types, such as breast, lung, prostate, renal cell, uterus, ovary and colorectal cancers, it has been well established that the genomic landscape of metastases is altered from that of the primary tumour.^9–11^ This is likely due to the development of certain molecular alterations that allow for a growth and invasion advantage to the metastatic cell.^8^ Next-generation sequencing has revealed the genetic heterogeneity between the primary tumour and metastatic deposits, between metastatic deposits from different sites, and even within different regions of the same tumour.^8^ This likely accounts for a major mechanism of treatment resistance and failure. Accordingly, in breast cancer, for example, guidelines suggest to biopsy metastatic sites to retest for oestrogen receptor, progesterone receptor and HER2 expression, which have primary tumour to metastasis discrepancy rates of 9–18%, 24–31% and 10%, respectively.^12^

However, unlike many other solid tumours, pancreatic cancer is a disease in which microscopic metastases likely develop early in the course of the disease, which would explain the high recurrence rate even in, for example, margin-negative, and local lymph node uninvolved surgically resected patients.^13^ These circulating metastases were likely already present at initial diagnosis, suggesting that key mutations occur early in pancreatic cancer development, before the tumour cells disseminate.^14^ This implies that the genetic architecture of a metastatic pancreatic cancer reflects that which is already present within the primary tumour.^15^

In an effort to test this theory, and to better understand the molecular landscape of primary versus metastatic pancreatic adenocarcinomas, we compared the frequency of genetic, protein and phosphoprotein alterations from primary and metastatic pancreatic tumour samples and from metastases of different sites. By focusing on potentially actionable information using real-world data, we explored whether targeted therapies could be tailored to patients at metastatic progression based on surgical material of the primary tumour.

## Methods

### Patients and tumour samples

Patients diagnosed with pancreatic cancer were enrolled through an IRB-approved registry protocol after being referred directly to the Perthera Precision Medicine program, or to the Know Your Tumor Program, a project run by Perthera and the Pancreatic Cancer Action Network to facilitate molecular testing of pancreatic cancer patients across the United States. Only patients with biopsy-confirmed pancreatic adenocarcinoma were included in the analysis cohort. Patients with pancreatic adenosquamous carcinoma or other histological subtypes were excluded from these analyses.

Tumour biopsy samples were sent to one or more CLIA-certified, CAP-accredited commercial laboratories for genomic, proteomic and/or phosphoproteomic testing. In general, formalin-fixed and paraffin-embedded blocks and/or slides were collected by surgical resection (e.g., whipple procedure or distal pancreatectomy), a core-needle biopsy or a fine-needle aspiration. When appropriate, samples were sectioned, randomised and co-mingled to minimise section-to-section cellular bias. Biopsies obtained within a year of testing were required for molecular profiling, though under certain circumstances, archived biopsies were used.

### Genomic profiling by next-generation sequencing (NGS)

A total of 713 patients received genomic profiling results that passed quality control measures (at least give genomic observations, including variants of unknown significance) to be included in these analyses. The filter was applied based on a population median of 12 genomic findings per patient, with 5th and 95th percentiles of 5 and 27, respectively. The majority of tumour tissue samples (689, 97%) were sent to Foundation Medicine (Cambridge, MA) for NGS testing of cancer-related mutations. Twenty-four patients (3% of total) received NGS testing results from Caris Life Sciences (Phoenix, AZ).

### Proteomic and phosphoproteomic profiling by immunohistochemistry (IHC)

Proteomic and phosphoproteomic profiling of up to 17 proteins was performed by immunohistochemistry (IHC). Tumour biopsy samples were sent to NeoGenomics (Fort Myers, FL) or Caris Life Sciences (Phoenix, AZ) for multiplexed IHC testing. Proteomic panels included potential markers of resistance to chemotherapeutic agents used in standard pancreatic ductal adenocarcinoma (PDAC) regimens, such as thymidylate synthase (TYMS or TS) for 5-fluorouracil,^16^ the DNA excision repair protein ERCC1 for oxaliplatin,^16^ topoisomerase 1 (TOP1 or TOPO1) for irinotecan,^17^ ribonucleoside diphosphate reductase (RRM1) for gemcitabine,^18^ and TUBB3 for taxanes.^19^ Protein-based markers associated with immunotherapies included PD-L1 (CD274), PD-1 (PDCD1) and the mismatch repair proteins MLH1, MSH2, MSH6 and PMS2. IHC markers associated with molecularly targeted agents included HER2 (ERBB2), MET and PTEN. A phosphoproteomic marker under development by NeoGenomics (Fort Myers, FL) that binds to phosphorylated AKT1/2/3 (pAKT) was also utilised.

### Tumour site-specific analyses

To investigate the potential limitations of interpreting molecular profiling results based on primary tumour specimens in patients with metastatic pancreatic cancer, we analysed the prevalence of molecular alterations across a cohort of 713 PDAC patients. The two most common sites of metastatic PDAC lesions used for molecular profiling were obtained from core liver biopsies and lung biopsies. Beyond the pancreas, non-liver lesions within the abdomen (e.g., retroperitoneum, duodenum, etc.) were also common and thus interpreted as a third category of metastatic sites. Metastatic lesions that did not fall within these three categories (e.g., central nervous system, heart) were excluded from these analyses.

### Quality control metrics

When estimating mutation frequencies in patient populations, tissue quality has a significant impact on the ability to detect genomic variants. When reduced sensitivity was noted by the genomic testing laboratory due to tissue sample quality, genomic profiles with fewer than five distinct genomic variants detected (of either known or unknown significance) were excluded from the analysis cohort (cut-off determined by visually inspecting a histogram of variant counts across patients).

### Comparative analyses of molecular findings

Summaries of molecular alterations (e.g., pathogenic variants, amplifications, copy-number deletions and oncogenic fusions) for individual genes were performed across patient cohorts based on the PDAC tumour site that was biopsied and tested. Cohorts included pancreatic primaries and metastatic liver, lung and abdominal lesions. An overall metastatic lesion cohort included liver, lung and abdominal lesions. Fisher’s exact test was used in our exploratory analyses comparing primary tumours with metastatic lesions. For site-specific comparisons, mutation frequencies were analysed between cohorts, using logistic regression models implemented in the R/Bioconductor programming environment. Protein and phosphoprotein expression data were analysed using a multifactor logistic regression model that included positive expression as a variable, as well as a term for the proteomic testing laboratory. Multiple hypothesis corrections were applied separately for proteomic and genomic observations for each comparison, and false-discovery rate-adjusted *q*-values were considered at a significance threshold of 0.05.

## Results

### Operational summary and baseline characteristics

Multi-omic molecular testing results were obtained from a cohort of 713 pancreatic adenocarcinoma patients who received a Perthera Report describing the actionability of their molecular findings. Actionability was defined by therapeutically applicable molecular subgroups. The majority of the molecular subgroups identified included mismatch repair gene deficiency (i.e., MLH1, PMS2, MSH2 and MSH6), homologous recombination repair (i.e., BRCA 1/2, ATM), cell cycle regulation (i.e., CDK 4/6) and HER2 amplification. These patients were referred from a wide range of high-volume tertiary cancer centres and community practices across the United States. The majority of patients were <65 years of age, and the ratio of male to female was equal (Table 1). The majority were originally diagnosed with metastatic disease (*n* = 336, 47%); however, some were diagnosed with disease only in the pancreas: either localised/resectable (*n* = 56, 8%) or locally advanced/borderline resectable (*n* = 321, 45%).

**Table 1:** Baseline patient characteristics and tumour location

	Primary (*n* = 282)	Metastatic (*n* = 431)
Gender		
Male	96 (53%)	172 (53%)
Female	86 (47%)	151 (47%)
Age		
<65 years old	155 (55%)	251 (58%)
>= 65 years old	127 (45%)	180 (42%)
Ethnicity		
African American	5 (2%)	7 (2%)
Ashkenazi Jew	3 (1%)	4 (1%)
Asian	10 (4%)	11 (3%)
Caucasian	130 (46%)	174 (40%)
Hispanic	10 (4%)	6 (1%)
Other	6 (2%)	7 (2%)
Not available	118 (41%)	222 (51%)
Tumour location	No	%
Primary	282	40%
Liver	278	37%
Lung	54	7%
Abdomen	99	13%

Comprehensive multi-omic (genomic, proteomic and phosphoproteomic) testing was performed by external laboratories for 713 patients with pancreatic adenocarcinoma (Table 1). Of these 713 patients, patient tumour samples that underwent molecular profiling were often biopsies or surgical samples of the pancreatic primary (*n* = 282, 40%) (Table 1). Outside the pancreas, molecular testing of liver biopsies was the most common (*n* = 278, 37%), followed by lesions in the lung or nearby in the thoracic cavity (*n* = 54, 7%). Molecular profiling of tumour samples within the abdomen but not in the liver or pancreas was also common (*n* = 99, 13%). Other lesions outside these areas (e.g., brain, heart) were also provided in a handful of cases (*n* = 32, 4%), but were ultimately excluded from the analyses presented in this study, due to small sample sizes across unrelated sites.

### Frequency of genetic alterations does not vary between primary and metastatic tumours

**Fig. 1:**
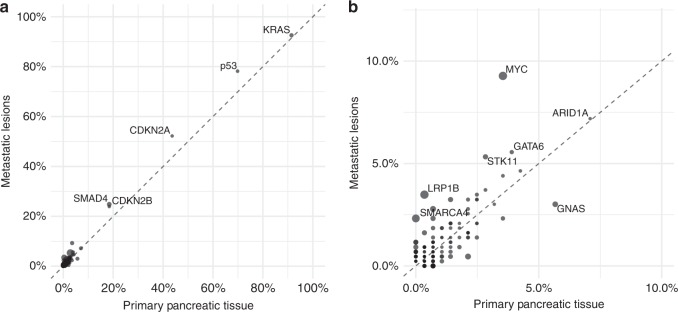
**a** Comparative frequencies of common mutations between metastatic lesions and primary pancreatic tumours. **b** Less common mutational frequencies between metastatic lesions and primary pancreatic tumours

To investigate the potential limitations of interpreting molecular profiling results based on primary tumour specimens in patients who currently have or may eventually develop metastatic pancreatic cancer, we analysed the prevalence of molecular alterations in primary and metastatic lesions. Comparative analyses of mutation frequencies between primary versus metastatic pancreatic adenocarcinomas revealed that the molecular features were similar across common genomic alterations, such as *KRAS*, *TP53*, *CDKN2A/B* and *SMAD4*, which were to be expected based on previous data^8^ (Fig. 1a). No statistically significant differences in specific genes were observed between primary versus metastatic lesions (false-discovery rate-adjusted *q*-value > 0.05). There were several genes with modestly higher frequencies in metastatic lesions prior to multiple testing correction: *MYC* (more frequent in metastatic lesions, unadjusted *p*-value = 0.005), a cell cycle regulator whose amplification is associated with poor prognosis in pancreatic cancer,^20^ and *LRP1B* (more frequent in metastatic lesions, unadjusted *p*-value = 0.025), a tumour suppressor involved in lipid processing (Fig. 1b). While these observations did not achieve significance that survived multiple hypothesis testing, these exploratory results warranted further investigation into possible subgroups.

**Fig. 2:**
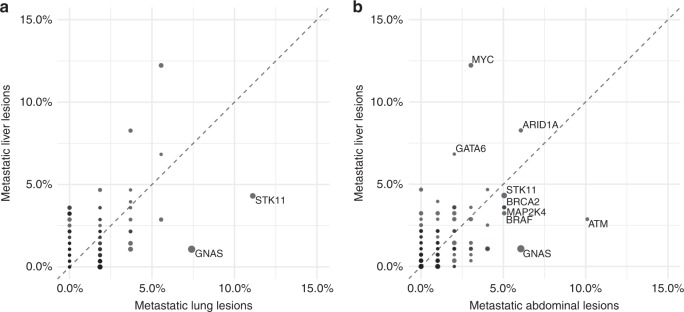
**a** Comparative frequencies of genomic alterations between metastatic liver and lung lesions. **b** Comparative frequencies of genomic alterations between metastatic liver and abdominal lesions

**Fig. 3:**
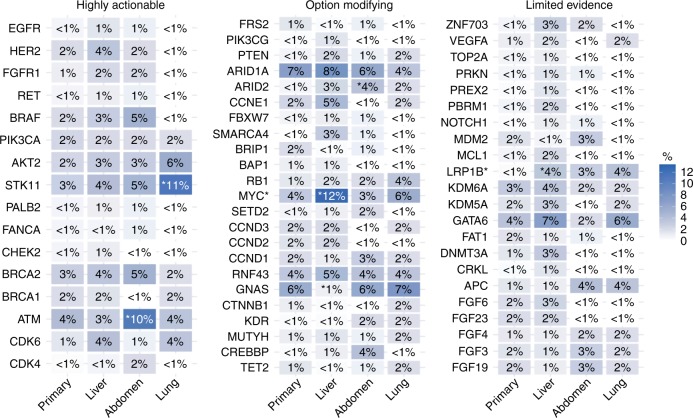
Frequency heatmap comparing actionability of molecular alterations within the primary tumour site compared with metastatic sites (liver, abdomen and lung)

To explore possible relationships between individual tumour sites and specific genomic alterations, we performed comparative analyses of mutation frequencies between primary tumours (*n* = 282), metastatic liver (*n* = 278), metastatic lung (*n* = 54) and metastatic abdominal (*n* = 99) lesions (Fig. 2a, b). In these analyses, we found that *MYC* alterations were significantly more common in liver lesions (12% compared with 3–6%) (Fig. 3) than in primary tumours (FDR-adjusted *q*-value = 0.004). There were no significant mutation variabilities between different individual metastatic sites after false-discovery rate correction. Genes with modest differences trending towards significance when comparing individual metastatic sites with primary tumours (Fig. 3) included STK11, a negative regulator of mTOR signalling via AMPK,^21^ which was mutated at a higher frequency in lung lesions (unadjusted *p*-value = 0.0097). Within abdominal lesions, two genes involved in DNA damage repair^22,23^ were slightly more prevalent: ATM (unadjusted *p*-value = 0.0373) and ARID2 (unadjusted *p*-value = 0.0424).

### Protein expression varies significantly between primary and metastatic tumours

**Fig. 4:**
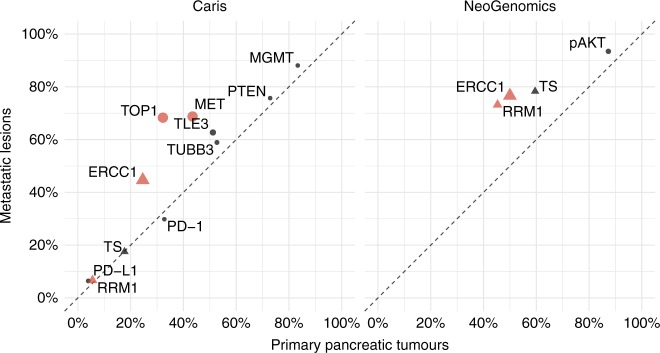
Comparative frequencies of protein and phosphoproteomic profiling between primary and metastatic tumours

We then compared proteomic results and found higher frequencies (false-discovery rate-adjusted *q*-value < 0.05, logistic regression) in ERCC1, TOP1 and MET protein expression in metastatic lesions compared with primary tumours (Fig. 4). For ERCC1 and RRM1 protein expression, these findings were significant even in the context of substantial laboratory-specific differences (Fig. 4).

**Fig. 5:**
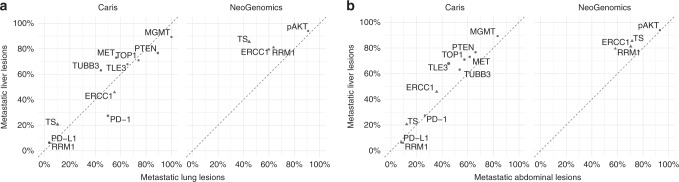
**a** Comparative frequencies of protein and phosphoproteomic positivity between metastatic liver and lung lesions. **b** Comparative frequencies of protein and phosphoproteomic positivity between metastatic liver and abdominal lesions

Within distant metastases, we observed possible tissue-specific patterns of protein expression (Fig. 5). The majority of the proteins analysed occurred with similar frequencies across site-specific mutations, with the exception of increased TUBB3 and decreased PTEN expression in the liver compared with the lung (FDR-adjusted *q*-value < 0.1).

### Actionability of mutations in pancreatic cancer

We next evaluated the frequency of actionable gene alterations (Fig. 3). Actionability was defined as the presence of one or more molecular alterations associated with potentially increased responsiveness to one or more therapeutic classes of agents.^24^ Biomarker associations were categorised into two tiers of actionability (highly actionable and option modifying), based on recommendations provided by expert oncologists who reviewed the molecular findings on a virtual molecular tumour board platform.^24^ We found no significant difference in the proportions of molecular profiles from primary tumours, with highly actionable genomic alterations, which was 62 (22%) versus 105 (24%) for metastatic lesions (*p*-value > 0.05, Fisher’s exact test). No significant differences were observed based on counts for individual metastatic sites, with 19% of lung, 24% of liver and 27% of abdominal lesions harbouring highly actionable alterations. Overall, these results suggest that molecular profiling of primary tumour tissue could potentially serve as an adequate substitute for a biopsy of the metastatic lesion; however, prospective studies with larger cohorts of patients with molecular testing results from matched primary and metastatic lesions are needed to confirm these findings. These studies should focus on the potential limitations of molecular profiling of surgical specimens, particularly for therapies with implications for the most commonly identified mutations (Fig. 3) within the DNA repair pathway (ATM, BRCA1 and BRCA2), the PI3K/AKT/mTOR pathway (PIK3CA, AKT2, STK11 and ARID1A), the MAPK pathway (BRAF), the Wnt signalling pathway (GNAS and RNF43) and the cell cycle pathway (CDK4, CDK6, CCND1, CCND2 and CCND3).

## Discussion

We compared the molecular (NGS exome and IHC/proteomic) characteristics of primary and metastatic pancreatic adenocarcinomas in patients who have received Perthera Reports in the context of CLIA/CAP-based targeted panels of genes and proteins that convey “actionable” information. Actionability was utilised in a subset of patients with pancreatic cancer from the Know Your Tumor Initiative and recently published.^24^ In brief, an integrated approach using precision medicine was feasible and resulted in improved progression-free survival when patients with an actionable alteration were matched to a targeted treatment. Actionability was defined if there was literature supporting clinical evidence of a high response rate in patients with that molecular abnormality in any cancer or a possible implication of response to therapy based on the underlying mechanism.^24^ In other solid tumours, previous studies have shown a substantial molecular discordance and heterogeneity that exists between primary and metastatic sites. As mentioned above, in breast cancer, a considerable number of tumours change their hormone receptor status at relapse or metastasis, thereby altering potential effective therapeutic strategies.^25^ In colorectal cancer, analysis of metastatic deposits revealed differences in specific mutations and protein expression, suggesting a molecular or pathway-driven treatment approach in place of, or in addition to, standard chemotherapy.^26^

In our study, we observed that, in PDAC, NGS-based actionable alterations were identified at the same frequency. While a few genes achieved significance, none were found to be significantly different after multiple hypothesis testing correction was applied. This was true regardless of how common a molecular alteration was in pancreatic cancer. In general, commonly mutated genes in pancreatic cancers did not substantially differ across tumour sites, with the exception of MYC, which was more frequent in the liver.

Our results are consistent with similar studies published recently.^27^ Makohon-Moore et al. applied whole-genome sequencing to multiple metastatic lesions from four pancreatic cancer patients and found the same driver gene mutations in every lesion. In addition, they also found similarities between non-driver genetic mutations, suggesting an inherent uniformity between primary and metastatic lesions.^28^ McDonald et al. went further to propose that although driver mutations occur early in pancreatic cancer tumorigenesis, epigenomic modifications likely aid in tumour invasion and metastatic spread.^29^ In addition, higher-impact driver mutations like frameshift or nonsense mutations are more commonly seen among all metastatic sites, while those that are not shared in all sites are less likely to have functional significance.^30^ Collectively, these studies further emphasise that a patient’s tumour sample can provide important genomic information, regardless of when (i.e., biopsy vs resection) or where (i.e., primary vs metastatic site) the specimen was obtained.

A potential limitation to our analysis was that the majority of primary and metastatic tissue samples were not obtained from matched individuals. This may account for heterogeneity of specific genomic and proteomic alterations, but based on our results, when looking at the genomic and proteomic actionable biomarker landscape at a frequency-based level across populations, our results indicated that NGS alterations were not affected, but the proteome appears to be. Out of the available tumour samples, primary and metastatic material was matched for three patients, without significant genomic differences (Supplementary Fig. 1a–c).

This work extends previous studies by directly comparing actionable molecular mutations in both primary tumour and metastasis-derived tissues that indicate potential therapeutic intervention.

Our data support the belief that primary pancreatic cancers metastasise very early and thus are indistinguishable from metastatic lesions at the actionable exome level.^31^ While genomic alterations were seemingly not different between primary versus metastatic sites, the levels of a number of protein-based chemopredictive markers did differ within the context of metastasis. This could be expected, given the impact of the tumour microenvironment on protein expression regulation and would represent an important finding for precision oncology-based workflows in the context of treatment recommendation efforts. At the pathway level, we found that therapeutically actionable molecular alterations were relatively consistent across primary and metastatic lesions.^24^ In addition, molecular profiling is increasingly used to match patients to targeted therapies, but obtaining a high-quality biopsy specimen from metastatic sites can be difficult. Moreover, the average survival of patients with pancreatic cancer is short even when enrolled onto clinical trials, with ~5% alive within a year of enrollment.^32^ This suggests that targeted therapies that are informed by NGS exome-based profiling could be provided, based on the initial surgical sample, which limits the need to re-biopsy at recurrence and decrease patient discomfort and anxiety, cost and delay to the next treatment. However, IHC/proteomic-based biomarkers that are used for treatment selection and patient selection would need to be carefully considered and validated in the context of metastatic tissue in light of these findings. In addition, the role of biomarker-directed therapy for early-stage pancreatic cancers in lieu of, or in addition to standard therapy, could be further evaluated in prospective clinical trials.

## Supplementary information

Supplementary Figures and Figure Legends

## Data Availability

Archival study data used to produce the results reported in this article can be found at Perthera and the Pancreatic Cancer Action Network’s Know Your Tumor program.
