# Supplementary material for: Multi-omic molecular comparison of primary versus metastatic pancreatic tumours
Source: Br J Cancer. 2019 Jul 11;121(3):264–70. doi: 10.1038/s41416-019-0507-5 (PMC6738081; doi:10.1038/s41416-019-0507-5)

**Supplementary Figure Legends:**

Supplementary Figure 1a - c: Genomic details for matched primary and metastatic material for three example patients.

**Figure 1a**


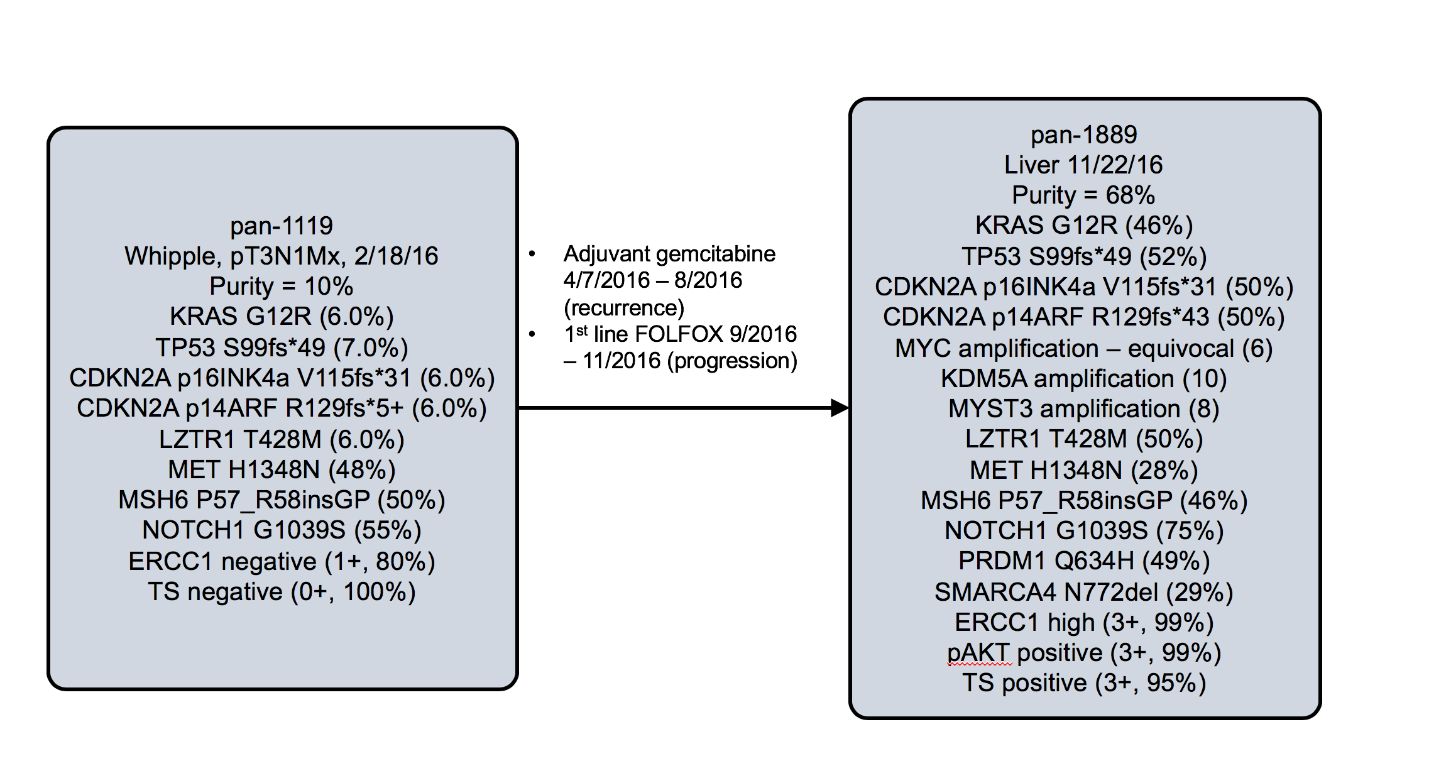


**Figure 1b**


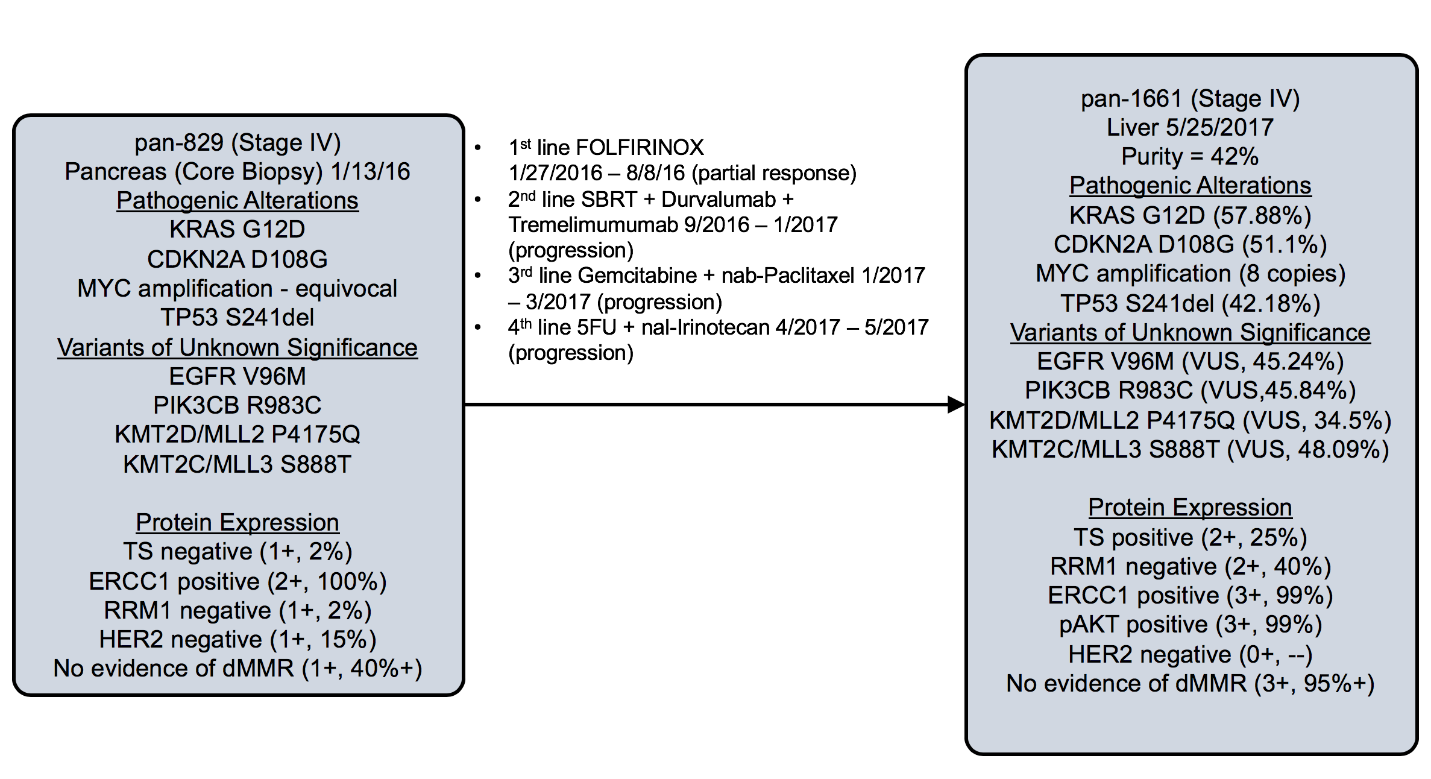


**Figure 1c**


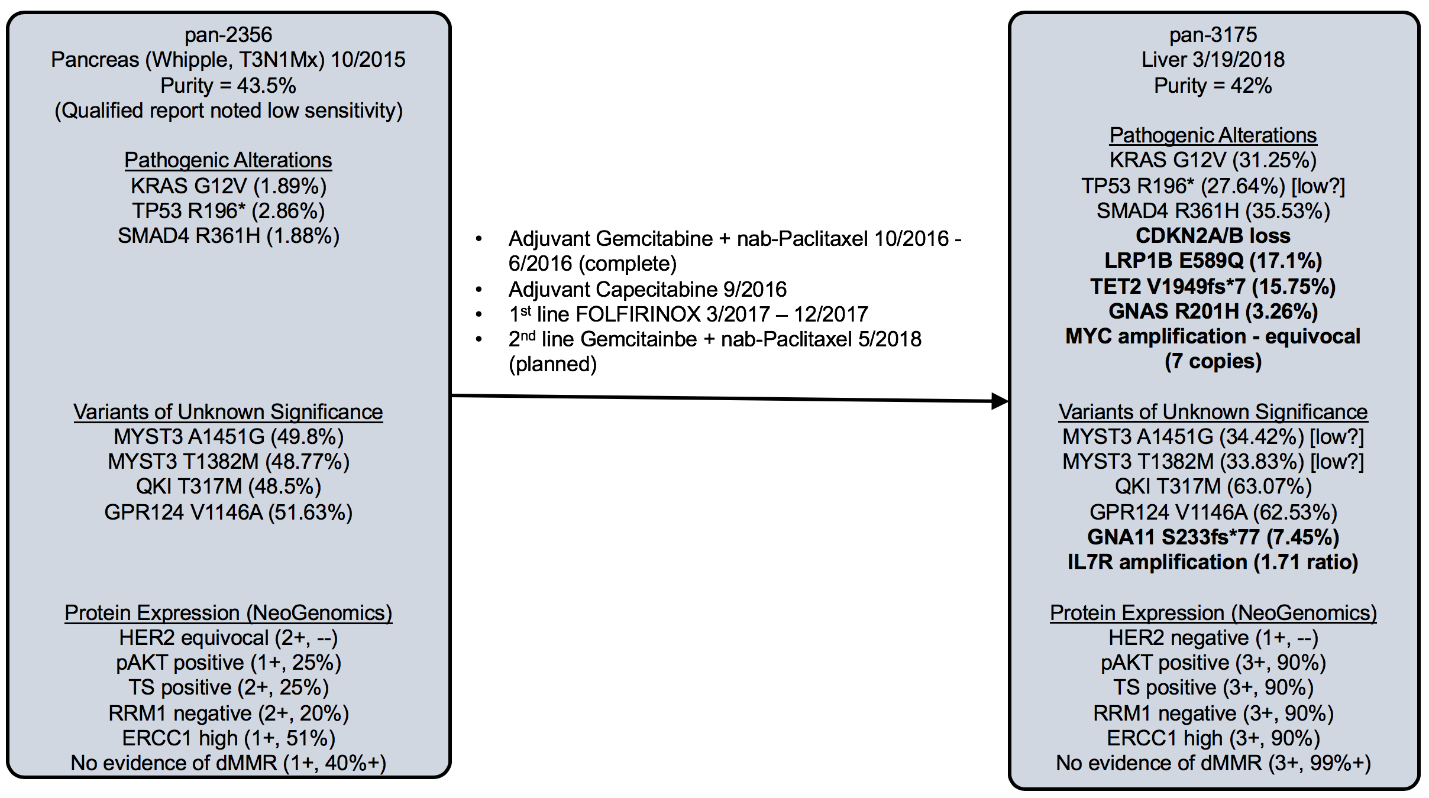

Supplement: Supplementary file 1 — Supplementary Figures and Figure Legends [file 41416_2019_507_MOESM1_ESM.docx]
